# Geographic information system for improving maternal and newborn health: recommendations for policy and programs

**DOI:** 10.1186/s12884-016-1199-y

**Published:** 2017-01-11

**Authors:** Yordanos B. Molla, Barbara Rawlins, Prestige Tatenda Makanga, Marc Cunningham, Juan Eugenio Hernández Ávila, Corrine Warren Ruktanonchai, Kavita Singh, Sylvia Alford, Mira Thompson, Vikas Dwivedi, Allisyn C. Moran, Zoe Matthews

**Affiliations:** 1 USAID’s Maternal and Child Survival Program/Save the Children, Washington, DC USA; 2 USAID’s Maternal and Child Survival Program/Save the Children, 14136 Grand Pre Rd #34, Silver Spring, MD Zip: 20906 USA; 3 USAID’s Maternal and Child Survival Program/Jhpiego, Washington, DC USA; 4 Geography Department, Simon Fraser University, Burnaby, BC Canada; 5 Department of Surveying and Geomatics, Midlands State University, Gweru, Zimbabwe; 6 MEASURE Evaluation/John Snow Inc, Rosslyn, VA, USA; 7 National Institute of Public Health of Mexico, Cuernavaca, Morelos Mexico; 8 Geography and Environment, University of Southampton, Southampton, UK; 9 MEASURE Evaluation/Carolina Population Center, University of North Carolina at Chapel Hill, Chapel Hill, NC USA; 10 Department of Maternal and Child Health, Gillings School of Global Public Health, University of North Carolina at Chapel Hill, Chapel Hill, NC USA; 11 Global Health Fellows Program II, United States Agency for International Development (USAID), Washington, DC USA; 12 USAID’s Maternal and Child Survival Program/ John Snow Inc, Washington, DC USA; 13 Department of Social Statistics and Demography, University of Southampton, Southampton, UK

**Keywords:** Maternal, Newborn, Mortality, GIS, Mapping

## Abstract

This correspondence argues and offers recommendations for how Geographic Information System (GIS) applied to maternal and newborn health data could potentially be used as part of the broader efforts for ending preventable maternal and newborn mortality. These recommendations were generated from a technical consultation on reporting and mapping maternal deaths that was held in Washington, DC from January 12 to 13, 2015 and hosted by the United States Agency for International Development’s (USAID) global Maternal and Child Survival Program (MCSP). Approximately 72 participants from over 25 global health organizations, government agencies, donors, universities, and other groups participated in the meeting.

The meeting placed emphases on how improved use of mapping could contribute to the post-2015 United Nation’s Sustainable Development Goals (SDGs), agenda in general and to contribute to better maternal and neonatal health outcomes in particular. Researchers and policy makers have been calling for more equitable improvement in Maternal and Newborn Health (MNH), specifically addressing hard-to-reach populations at sub-national levels. Data visualization using mapping and geospatial analyses play a significant role in addressing the emerging need for improved spatial investigation at subnational scale. This correspondence identifies key challenges and recommendations so GIS may be better applied to maternal health programs in resource poor settings. The challenges and recommendations are broadly grouped into three categories: ancillary geospatial and MNH data sources, technical and human resources needs and community participation.

## Background

This article offers recommendations for how mapping and Geographic Information System (GIS) applied to maternal and neonatal health data could potentially be used as part of the broader efforts for ending preventable maternal and newborn mortality. These recommendations were generated from a technical consultation on reporting and mapping maternal and neonatal deaths that was held in Washington, DC from January 12 to 13, 2015 and hosted by the United States Agency for International Development’s (USAID) global Maternal and Child Survival Program (MCSP) Approximately 72 participants from over 25*^1^ global health organizations, government agencies, donors, universities, and other groups participated in the meeting.

The meeting placed emphases on how improved use of mapping could contribute to the post-2015 United Nation’s Sustainable Development Goals (SDGs), agenda in general and to contribute to better maternal health outcomes in particular. The Millennium Development Goal of reducing maternal mortality ratio by 75% (MDG5) was not achieved by the 2015 deadline, despite substantial progress toward advancing the health and well-being of women over the past decade. Global focus has now shifted to achievement of the SDGs which similarly propose to improve maternal health and reduce mortality to less than 70 per 100,000 live births by and reduce neonatal mortality to at least 12 per 1,000 live births in 2030 [1]. Achieving these goals will require national maternal and newborn health (MNH) programs to address underlying, localized inequalities [2]. Researchers and policy makers alike are therefore calling for more equitable improvement in MNH, specifically addressing hard-to-reach populations at sub-national levels [3].

Data visualization using mapping and geospatial analyses play a significant role in addressing the emerging need for improved spatial investigation at subnational scale through 1) mapping key MNH service provision indicators as well as associated determinants; 2) analyzing geographic access to MNH services e.g., access to emergency obstetric care (EmOC); and 3) modeling potential actions to identify how best to increase such access to maternal and neonatal health services [4]. This correspondence identifies key challenges and recommendations so GIS may be better applied to maternal health programs in resource poor settings. The challenges and recommendations can be broadly grouped into three categories: 1-ancillary geospatial and MNH data sources, 2-technical and human resources needs and 3- community participation.

## Ancillary geospatial and MNH data sources

Critical to any geospatial enquiries at national or sub-national level are accurate, up to date, and reliable geospatial data including both ancillary geospatial data and health data. Ancillary data includes population estimates, subnational boundaries, roads and rivers, etc. Health data can be separated into non-routine data such data from surveys, and routine data (e.g., from health information systems, health facility registries, maternal death surveillance and response systems, and vital registry systems). Whether examining access to health facilities or predicting skilled birth attendance, the local environment plays a critical role in influencing MNH. As such, close attention must be paid to the types of databases used in analysis, as well as geographical division and time of data collection.

The DIVA-GIS project, is a commonly used, consolidated source of country-level and global ancillary data that is freely available [5]. Information on the distribution of populations is also freely available through the WorldPop project (www.worldpop.org) including high-resolution data on human population distributions for countries in Africa, Asia, and Central and South America [6]. Specifically in the context of MNH, distributions of live births, pregnancies and women of childbearing age are available both on the 100-meter level and administrative unit 2 level, where applicable, as well as important covariates including poverty, literacy, and urban change. The methods used to generate distributions of live births and pregnancies have been described in detail in the literature [7], and have been used in a variety of publications, including peer-reviewed literature and policy reports, such as the UNFPA’s 2014 State of the World’s Midwifery Report [8].

Non-routine survey data, such as from the Demographic and Health Surveys (DHS), provide a rapid entry into the use of GIS for MNH. DHS data provides users with a readily accessible, freely available source of geo-located household and facility-based surveys which can be used to model an array of MNH outcomes, both within and across countries [9]. The DHS allows researchers to link household survey data with national health facility [10] data, such as those from the Service Provision Assessment (SPA) also conducted by the DHS international survey program. DHS data are of high quality and widely used throughout the literature, however, recent critical appraisals have identified common inconsistencies such as definition of skilled birth attendants and challenges inherent to administering the survey across low and middle income countries, and call for caution when employing inter-country DHS data specifically in the context of MNH [11]. Surveys are often conducted every 5 to 10 years-not frequently enough for general program monitoring. Additionally, maternal mortality-a key indicator-can usually only be mapped at the national level because the commonly used sisterhood methods does not record location of the deaths for making subnational estimates [12, 13]. Spatial resolution from surveys has limited their use to the state or province level, though recent guidance [14] on interpolated maps using DHS data may lead to new avenues for detailed spatial investigation. Neonatal mortality-another key indicator-could also potentially be mapped at a sub-national level, depending on the sample size of the survey.

Verbal autopsy is another data source that can be used to assess cause of death by geographic area. As many deaths occur outside of the formal health care system, verbal autopsy is used to assess the cause of death after either a household survey or after a census. Verbal autopsies have also been used as part of Demographic Surveillance Sites, Sample Registration Sites in India and Disease Surveillance Points in China to ascertain major causes of death in a defined geographic area. The WHO has a manual on verbal autopsies with sample questionnaires included [15], and the latest International Classification of Disease (ICD 10) guidelines are typically used to assign cause of death [16, 17].

Other comprehensive, yet less readily available sources of MNH data include country-specific census data and emergency obstetric and newborn care (EmONC) surveys data [18]. In the 2010 census round, 21 African, 10 Asian, 6 Latin American and 1 Oceania countries collected data on pregnancy-related mortality in the national census. Questions included in the census are intended to ascertain if any women of reproductive age had died during pregnancy or within 6 weeks after giving birth. Census data yields information on pregnancy-related deaths (all deaths occurring during pregnancy and the postpartum period) rather than maternal deaths (deaths occurring during pregnancy and up to 6 weeks postpartum from any cause related to or aggravated by the pregnancy or its management, but not from incidental causes). As a result, true maternal deaths may be overestimated; however, this overestimation may be compensated by the fact that some pregnancy-related deaths (e.g., from induced abortion) may not be reported. In addition, the number of reported births and deaths during a census is usually biased (upwardly skewed in both census and surveys) because the questionnaire asks about pregnancy-related deaths rather than actual maternal deaths [19, 20]. Moreover, access to these data sources can prove challenging, because they are typically housed within country-level ministries of health or other government agencies rather than the public domain. Therefore strong partnerships should be set up between researchers and program officers. Regardless, comprehensive datasets such as census data or vital registries are ideal for measuring rare events, such as maternal mortality, as they allow for more accurate estimation of events [21].

Routine health information systems such as HMIS, Maternal Death Surveillance and Response (MDSR), and Civil Registration and Vital Statistics (CRVS) address the challenges of temporal frequency and geographic scale, yet face their own challenges [22]. In recent years, substantial investments have been made in health management information systems in many low and middle income countries. One of the health management information systems that has been increasingly adopted is District Health Information System 2 (DHIS2). These systems have shown promising results in reporting of aggregate data to higher levels and include a platform for data visualization. Expansion of any electronic platform at health facility level, however, requires improved electronic infrastructure at country level. Standardization of reporting will help in better integration of maternal, perinatal and neonatal death reporting system between multiple data sources. This will facilitate easy availability of coverage and mortality data and promote routine use of such data for mapping and thus provide health workers and decision-makers with the tools that facilitate decision-making.

The Maternal Death Surveillance and Response (MDSR) guidelines developed by the WHO provide a standardized framework for establishing national protocols for reporting and action on maternal deaths and may help improve the availability and quality of maternal death data in low and middle income countries moving forward. The primary objective of MDSR is to provide information that effectively guides actions to eliminate preventable maternal mortality and count every maternal death, permitting an assessment of the true magnitude of maternal mortality and the impact of actions taken to reduce it [23]. Locally generated routine national and subnational estimates are more relevant for decision making than periodically generated global or national estimates. Therefore, a routine data source such as MDSR will provide better measurement and information for action to prevent maternal deaths at local, health facility and district levels, and in sensitizing communities and facility health workers, and creating country ownership of real time data [23, 24]. Adoption of MDSR into national protocol should take into consideration operationalizing MDSR for geospatial mapping at different levels. In addition, a perinatal death tool is in process to be incorporated with MDSR [25].

There is broad consensus that improving mortality statistics is central to building more comprehensive CRVS systems in the post-2015 era [25]. The WHO consultation from November 2014 identified four prongs of action for the weakest systems (death registration coverage below 60%) as part of an iterative approach to improving mortality statistics: CRVS platform development, innovation, health facility-based mortality statistics, and optimizing data from multiple sources [26]. Mapping maternal and neonatal deaths and taking the time to carefully design systems that incorporate GIS data will help achieve several of the proposed SDGs [27]. The authors noted that improved and geographically coded maternal and neonatal mortality statistics including GIS data will only lead to attainment of the SDG goals if the following occur:

1. GIS coded data is appropriately analyzed and fed back into a quality improvement cycle. This will ensure that learning from preventable maternal deaths is incorporated into future service delivery and providers are able to acknowledge mistakes without fear of retribution. Confidential inquiries are key to this process.
2. Inequities in the number of deaths between geographic locations are carefully analyzed at regular intervals and resources are appropriately targeted to foster improvements by managers and policy makers within the health systems.
3. There is careful consideration of incorporating data from private providers as well as the public health system to ensure that all maternal deaths are captured and registered.
4. At present, it should be noted that the SDG goals explicitly mention birth registration: “by 2030 provide legal identity for all including birth registration”. Birth registration along with mortality registration form part of a more comprehensive CRVS systems but require special attention.

## Key technical and human resources recommendations for producing high quality action-oriented maps of maternal health data in low resource settings

**Fig. 1:**
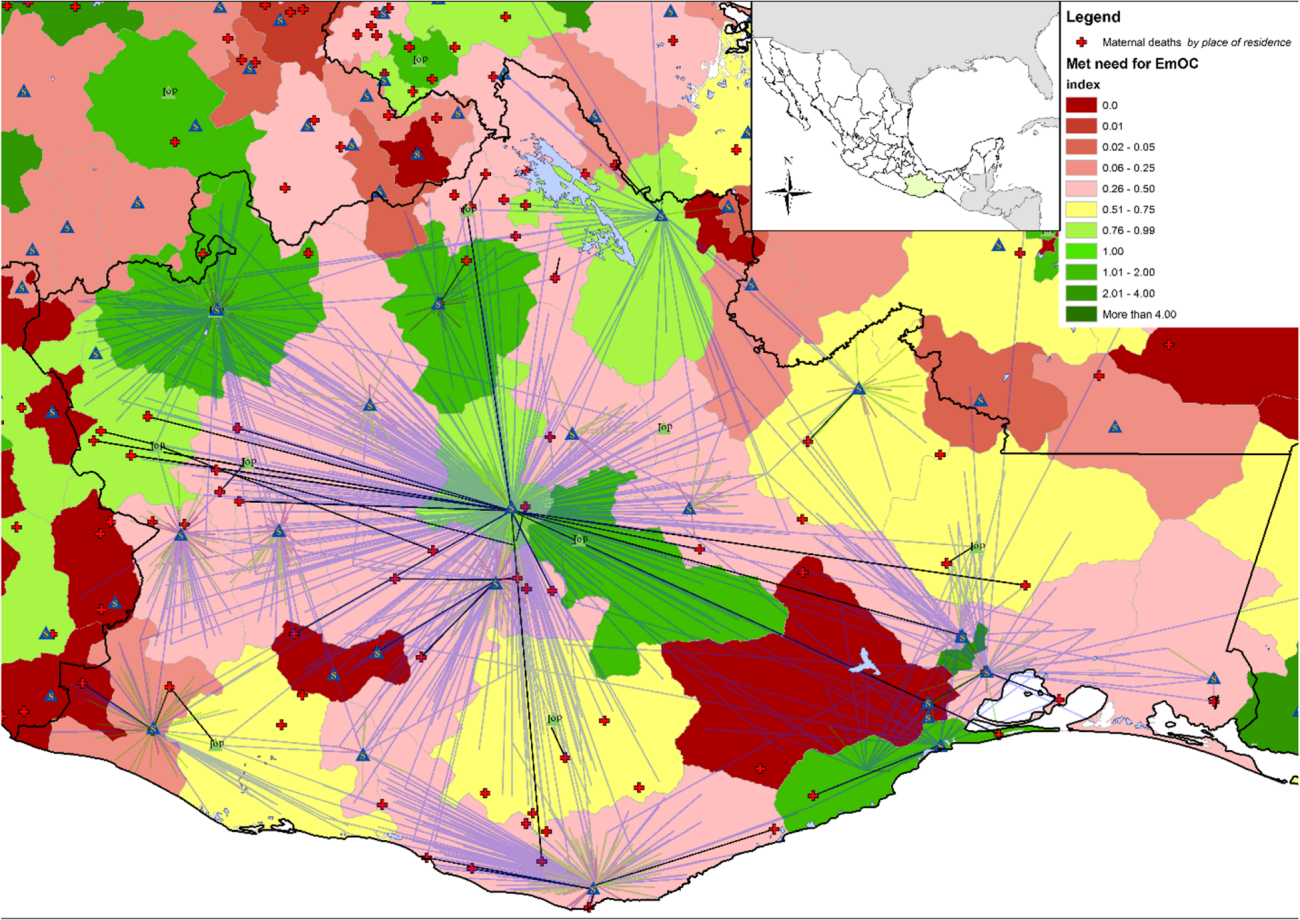
Origin-Destinations map of women seeking birth care services in the state of Oaxaca, Mexico 2007. This map was prepared by authors using public vital statistics (1), health facilities (2), hospital discharge (3) and demographic (4) data published by the Ministry of health and the National Institute of Satistics and Geography of Mexico [38–41]

One key technical recommendation that was emphasized during the technical consultation meeting was a need for mapping beyond maternal mortality. That includesspatial distribution of life saving interventions such as access to emergency services and skilled birth attendants, in addition to mapping maternal mortality. Previous and ongoing work with geographical approaches related to the health and survival of women has been presented by Ebener et al. [4]. Beyond mapping mortality distribution, GIS has largely been used for monitoring and reporting progress of maternal health interventions and quantifying access to facilities and care [4]. An example for utilizing GIS for non-mortality mapping has linked the State of the World’s Midwifery findings with GIS to ensure fair distribution of services and priority for the worst off [8]. Similarly, Mexico’s experience of mapping met-need for EmOC (an indicator that measures the proportion of women who need emergency obstetric care that actually receive it) at micro regional scales helps identify geographic areas of low utilization. This has facilitated identification of geographic areas that may require attention or further investigation in order to ensure use of quality EmOC services (Fig. 1). Mapping availability, accessibility and coverage of EmOC in Mozambique, Ethiopia, Ghana, Burkina Faso, Cambodia, Laos, Malawi, Philippines are some of the ongoing activities at present [4]. Other countries such as Bangladesh and Haiti have used GIS not only to map out facilities and compare need with services, but also to create a live geographically referenced system that monitors quality and results of local and national health services in real time [28, 29].

In addition to high quality data, the institutional capacity to perform geospatial analyses, including efforts to improve human resource availability for mapping maternal and newborn data in low and lower-middle income countries are needed. Yet while the past decade has seen substantial growth in the availability of free and open source software and high resolution geospatial data, human resource constraints remain a challenge limiting the use of geospatial tools in low and lower-middle income countries. Current global capacity building efforts targeted at low/low-middle income countries include developing mapping training curricula and building mapping capacity of local decision makers, facilitating practical sessions with mentorship and peer learning, linking maternal and newborn health programs with national statistics and mapping agencies or other sectors with GIS capacity, and supporting access to and utilization of user-friendly mapping tools that allow decision makers to routinely view their data from spatial perspectives. Some of the global responses for mapping capacity building include initiatives by DHS and MEASURE Evaluation. They have developed sets of guidance documents for using geospatial data for global health and training material for open source QGIS software. MEASURE Evaluation has also created a self-directed course on using geographic approaches to manage, analyze, and leverage spatial data effectively when planning, monitoring, and evaluating health sector programs [30]. WHO has developed a software that operates on ArcGIS platform to assess access to services (AccessMod).

## Community participation

It is known that both the development and use of high quality maps should involve community engagement and participation [31, 32]. Recent practices of participatory mapping have facilitated monitoring real-time data for mapping, supported interpretation of spatial analysis results, and fostered ownership and decision making by the communities engaged [31, 33]. The significant roles of community health workers and volunteers that improve health service delivery in low and middle income countries have the potential to be utilized for mapping maternal and neonatal health information [34]. Involving community health workers in mapping is also more cost effective and facilitates local decision making [35, 36]. Most importantly, participatory mapping of maternal and newborn data that is openly communicated with the public throughout the process ensures social accountability and continuous feedback.

Participatory mapping through the web offers great potential for creating some of the higher resolution community level ancillary data like roads that can be used for assessing access to maternal health services. This will still require some training for community stakeholders, who could potentially be community health workers, or other personnel living or working in the communities being mapped. Open mapping standards and technologies that allow for these participatory processes already exist [37] and could be used to enhance the poor spatial data infrastructures that characterize most low and middle income countries.

## Conclusion

**Fig. 2:**
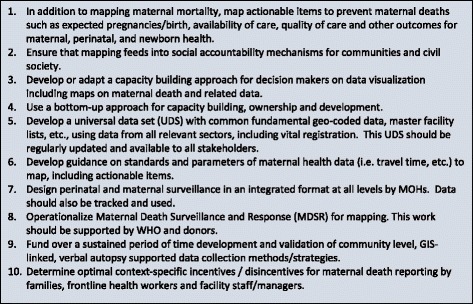
Top 10 recommendations for maternal mortality mapping that will have the greatest impact

In summary, the January 2015 technical consultation meeting discussed the future priorities and recommended actions for using GIS to contribute to better maternal health outcomes with ten top recommendations summarized under Fig. 2. The meeting highlighted that GIS holds substantial potential for supporting efforts to end preventable maternal and newborn deaths. Realizing this potential will require improved access to high quality MNH data at needed resolutions for decision makers at multiple levels, increased understanding of and skills in using both the software and the maps for planning and implementing MNH programs, and consistent involvement of the community-in the mapping process as well as in the use of high resolution maps. The MNH Network is an informal group of professionals working in data visualization for maternal and newborn health. This network will continue to coordinate and collaborate to ensure this work is incorporated into the post-2015 development agenda.

## Abbreviations

CRVS: Civil Registration and Vital Statistics
DHIS2: District Health Information System 2
DHS: Demographic and Health Surveys
EmOC: Emergency Obstetric Care
EmONC: Emergency Obstetric and Newborn Care
GIS: Geographic Information System
HMIS: Health Management Information Systems
ICD: International Classification of Disease
MCSP: Maternal and Child Survival Program
MDG5: Millennium Development Goal
MDSR: Maternal Death Surveillance and Response
MNH: Maternal and Newborn Health
QGIS: Quantum GIS
SDG: Sustainable Development Goal
SPA: Service Provision Assessment
UNFPA: United Nations Population Fund
USAID: United States Agency for International Development’s
WHO: World Health Organization

## Footnotes

1 List of organization that participated in the technical meeting: African Union Commission AMDD, Mailman School of Public Health, Columbia University, Children’s Investment Fund Foundation, Data Act Lab AB, Facultad de Medicina, Universidad de Antioquia, FHI 360, Independent, INDEPTH Network, Jhpiego, LSHTM, MCSP (partners from JSI, JHPIEGO, ICF), UNC Chapel Hill, MHTF, NICHD, NIH, Population Council, Simon Fraser University, The Demographic and Health Surveys Program, UNFPA, UNICEF, University of Aberdeen, University of British Columbia, Vancouver Canada, University of Southampton, University Research Co. LLC/USAID ASSIST Project, USAID/USAID GeoCenter, WHO/PAHO.
